# Navigating smoking cessation in healthcare: a pilot study of the SMOKE AKAT questionnaire among family medicine residents

**DOI:** 10.3389/fpubh.2025.1471124

**Published:** 2025-10-09

**Authors:** Iva Petričušić, Marko Marelić, Ljiljana Ćenan, Hana Brborović, Ognjen Brborović

**Affiliations:** ^1^Private Family Medicine Practice, Ivankovo, Croatia; ^2^School of Medicine, University of Zagreb, Zagreb, Croatia; ^3^Department of Medical Sociology and Health Economics, Andrija Štampar School of Public Health, School of Medicine, University of Zagreb, Zagreb, Croatia; ^4^Department of Environmental and Occupational Health and Sports Medicine, Andrija Štampar School of Public Health, School of Medicine, University of Zagreb, Zagreb, Croatia; ^5^Department of Social Medicine and Organization of Health Care, Andrija Štampar School of Public Health, School of Medicine, University of Zagreb, Zagreb, Croatia

**Keywords:** smoking cessation, family practice, health knowledge, attitudes, residency, education

## Abstract

**Introduction:**

Family medicine doctors play a crucial role in smoking cessation efforts but often lack adequate training and skills in this area. This study aimed to assess attitudes, knowledge, and behaviors regarding smoking cessation among family medicine residents using a newly developed instrument.

**Methods:**

A novel 29-item questionnaire called “Attitudes and Knowledge Assessing Tool on Smoking Cessation Methods” (SMOKE AKAT) was developed and administered online to 161 family medicine residents at the University of Zagreb. This cross-sectional survey assessed knowledge about smoking-related health risks, cessation methods, and harm reduction approaches, as well as attitudes and behaviors related to smoking cessation counseling. Descriptive statistics were used to analyze the responses.

**Results:**

93 residents completed the survey (57.76% response rate). Key findings include: 91.4% had never received formal education on smoking cessation methods; 62.4% incorrectly believed nicotine causes cancer; 84.9% incorrectly classified e-cigarettes as nicotine replacement therapy; only 57% correctly identified the definition of harm reduction; 51.6% reported spending 2–5 min on cessation counseling per patient visit; and 84.9% believed family doctors should be responsible for implementing smoking cessation interventions.

**Conclusion:**

In light of the European Commission’s agenda to make the EU smoke-free by 2040, where healthcare professionals play an increasingly crucial role this study revealed significant knowledge gaps and misconceptions about smoking cessation among family medicine residents. Many lack formal training but express interest in further education. There is a need to enhance smoking cessation curricula in family medicine training programs, focusing on evidence-based cessation methods, pharmacotherapy options, and harm reduction approaches. Improving residents’ knowledge and skills in this area could lead to more effective smoking cessation interventions in primary care settings. The SMOKE AKAT questionnaire addresses a critical gap in the current healthcare research landscape by providing an assessment tool to identify knowledge deficiencies, attitudinal barriers, and practice gaps among family medicine physicians in smoking cessation counseling and enables targeted educational interventions to correct specific deficiencies that might otherwise remain undetected in standard medical education assessments.

## Introduction

Changing harmful habits in the population remains a central challenge in public health, with family, educational systems and primary health medicine serving as the important focus of prevention strategies (1–7). While preventing the initiation of harmful behaviors is the ideal, once established, interventions rely heavily on the broad reach of primary healthcare and family medicine (6, 7). Although cultural and social norms have deeply embedded smoking in many societies, often granting it symbolic or social significance, these factors create substantial barriers for health professionals tasked with promoting smoking cessation (8, 9). Family physicians must address both physiological addiction and entrenched social influences, making the delivery of effective smoking cessation support particularly complex (8, 9). Therefore, specialized interventions and comprehensive approaches within primary care are needed (10, 11). Despite widespread awareness of smoking’s health risks, information alone rarely leads to behavioral change (9). Instead, targeted interventions by healthcare providers—who understand both the physical and social dimensions of smoking—are essential (8, 11). Primary care settings are recognized as the most effective environment for addressing tobacco dependence, thanks to their accessibility, continuity of care, and established patient-provider relationships (11–13). Moreover, their regular contact with diverse patient populations allows them to identify smokers, understand individual contexts, and provide personalized cessation support (11). Family medicine doctors, as frontline providers, are strategically positioned to support smoking cessation through ongoing patient relationships and personalized care (8, 13). However, despite this opportunity, the implementation of smoking cessation interventions in primary care remains inconsistent (12). Professional discourse reveals that methods for quitting smoking are often insufficiently discussed, and cessation counseling skills are frequently assumed rather than systematically developed (14). This assumption contrasts with evidence showing that many family medicine physicians lack the specific skills, knowledge, and attitudes needed for effective cessation support (9, 10).

The complexity of tobacco addiction—combining physiological dependence with behavioral and social factors—demands approaches that may exceed standard medical training (8, 10). Primary care providers are expected to be knowledgeable about evidence-based cessation methods and to assist patients in selecting and implementing appropriate strategies (8, 12, 15). Yet, research indicates that many physicians feel unprepared or uncomfortable discussing smoking cessation, citing concerns about damaging patient relationships, doubts about intervention effectiveness, or lack of training in motivational communication (9, 16, 17). Despite these challenges, the potential impact of primary care intervention remains substantial due to the reach and continuity of care in this setting (8, 12, 13).

International guidelines, including those from the World Health Organization, call for integrating smoking cessation services into primary healthcare systems and recognize the unique effectiveness of patient-centered approaches in primary care (13, 18). However, the persistent gap between the cultural entrenchment of smoking and the expectations placed on primary care highlights the need for enhanced training, resources, and systems to support family physicians (14, 16, 19).

In practice, many primary healthcare physicians perceive their role as one of detecting smoking-related health issues and encouraging cessation, often viewing quitting as a matter of personal willpower (20). This perspective may overlook the complexities of addiction and the need for a holistic approach, including harm reduction, addressing social determinants, and supporting patients with comorbidities. While the 5As framework (Ask, Advise, Assess, Assist, Arrange) guides comprehensive cessation support, gaps persist in moving beyond initial advice to providing ongoing assistance and follow-up, with only a minority of smokers receiving support beyond simple advice (19, 21, 22). Training health professionals in smoking cessation techniques has been shown to increase the likelihood of advising patients to quit (23–25). However, studies from various countries reveal that even when professionals feel knowledgeable, routine documentation and support for cessation—especially among vulnerable groups—remains limited (26). This global discrepancy between the provision of advice and the actual knowledge and skills about cessation methods is well documented (12).

In Croatia, where tobacco use rates are among the highest in Europe, family medicine doctors play a pivotal role in cessation efforts (27). However, significant gaps exist between the understanding of interventions and their implementation, as seen in both nursing and medical student populations, where formal training in cessation methods is rare (28, 29). These shortcomings in education and practice highlight the urgent need for improvement, despite Croatia’s history of tobacco control initiatives (30).

## Study aim

Despite the critical role of family medicine physicians in smoking cessation efforts, there is a significant gap in standardized instruments to assess their knowledge, attitudes, and behaviors regarding smoking cessation interventions. Current literature highlights that many healthcare providers lack formal training in cessation methods, yet are expected to effectively counsel patients—a disconnect that may contribute to suboptimal cessation outcomes. A validated assessment tool would address this gap by providing a systematic method to evaluate healthcare providers’ preparedness to deliver evidence-based smoking cessation interventions. The SMOKE AKAT questionnaire, whose novelty lies in its tailored focus on the specific educational, cultural, and systemic context, has been developed specifically to identify knowledge gaps, misconceptions, and attitudinal barriers that may impede effective cessation counseling. By creating and validating this instrument, researchers can establish baseline measurements of provider competencies, inform targeted educational interventions, track improvements over time, and ultimately contribute to public health policy development. The tool’s potential to standardize assessment across different healthcare settings would enable more consistent training approaches and facilitate comparisons between different educational strategies, thereby enhancing the effectiveness of smoking cessation efforts in primary care settings. Importantly, the SMOKE AKAT is not only a research tool but also has practical applications for training as an extension for national or WHO clinical treatment guideline for tobacco cessation in adults as it can establish baseline measurements of provider competencies, inform the design of targeted educational interventions, track improvements following training. By integrating the questionnaire into medical education programs, educators can tailor curricula to address specific deficiencies, monitor the impact of educational strategies, and support the European Commission’s goal of a smoke-free EU by 2040.

Therefore, this study aims to test the newly developed SMOKE AKAT instrument, evaluate specific attitudes, knowledge, and behaviors of family medicine residents, and consider the instrument’s potential value for public health policy and intervention strategies.

## Methods

### Study design

A cross-sectional design was used and quantitative data were collected through an online survey questionnaire (July 3–30, 2023) administered via Typeform®. A convenience sample was gathered from all 161 participants in the postgraduate study of the national family medicine specialist program.

### Measures

A new questionnaire named the “Attitudes and Knowledge Assessing Tool on Smoking Cessation Methods” (SMOKE AKAT) was constructed as a novel tool for assessing attitudes and knowledge among family medicine residents (Supplementary file 1). The questionnaire was developed based on a review of relevant literature (Supplementary file 2). The content validity of the questionnaire was established through the expert contributions of the authors, who are specialists in various but complementary fields relevant to this study: a public health specialist, a family medicine resident, an occupational and sports medicine specialist, and a sociologist. It consisted of twenty-eight questions, preceded by informed consent, research details, and researcher information, using the opt-out method. Participants who did not confirm informed consent did not participate in the study.

The questionnaire does not include complex measures such as scales; however, some questions can be thematically grouped into batteries based on what they assess. Accordingly, the instrument is structured around three key themes: (A) knowledge about smoking cessation and smoking in general (altogether 16 questions; questions number 1, 2, 6, 7, 8, 9, 10, 11, 12, 13, 14, 15, 16, 23, 24 and 26), (B) attitudes and behaviors (including self-assessed experiences and practices related to smoking cessation) (10 questions; questions number 3, 4, 5, 17 18, 20, 21, 22, 25 and 29), and (C) demographics (3 questions; questions number 19, 27 and 28). The key variables from these batteries are presented in Tables 1–6.

**Table 1:** Respondents’ answers regarding knowledge about smoking cessation and smoking in general.

	No		Yes		Third option	
	*N*	%	*N*	%	*N*	%
Substances believed by respondents to influence the occurrence of smoking-related cancers						
Inhalation of substances produced by the combustion of tobacco	5	5.4	88	94.6	N/A	N/A
Inhalation of substances produced by the combustion of cigarette paper	37	39.8	56	60.2	N/A	N/A
Inhalation of flavors (menthol/forest fruits etc.) added to cigarettes	37	39.8	56	60.2	N/A	N/A
Inhalation of various additives to tobacco used in cigarette preparation	6	6.5	87	93.5	N/A	N/A
Inhalation of nicotine	35	37.6	58	62.4	N/A	N/A
Abrupt smoking cessation is a better method than gradual withdrawal from smoking	31	33.3	38	40.9	24^a^	25.8^a^
Physical activity can contribute to achieving and maintaining smoking cessation	1	1.1	83	89.2	9^b^	9.7^b^
Group counseling or “schools of smoking cessation” is the best method for smoking cessation	23	24.7	70	75.3	N/A	N/A
Using mobile applications can increase the proportion of people who quit smoking long-term	30	32.3	63	67.7	N/A	N/A
Choose the medications you would prescribe to a patient as pharmacotherapy options for smoking cessation^c^						
Wellbutrin ®	60	64.5	33	35.5	N/A	N/A
Varenicline ®	50	53.8	43	46.2	N/A	N/A
Nicorette ®	22	23.7	71	76.3	N/A	N/A
Zomig ®	86	92.5	7	7.5	N/A	N/A
I would not prescribe medications as an option for smoking cessation	78	83.9	15	16.1	N/A	N/A
Mindfulness methods (yoga, Dialectical Behavior Therapy DBT, Acceptance and Commitment Therapy ACT etc.) contribute to smoking cessation	no clear benefits or evidence		extremely contribute		significantly contribute	
	*N*	%	*N*	%	*N*	%
	33	35.5	2	2.2	58	62.4

^a^The third option was: “There is no difference”.

^b^The third option was: “Slightly contributes”.

^c^Respondents could select multiple offered medications.

**Table 2:** Respondents’ rankings of successfulness of smoking cessation methods.

Smoking cessation method	Mean rank	Ranked 1st		Rank 2nd		Rank 3rd	
		*N*	%	*N*	%	*N*	%
Nicotine replacement therapy NRT	1.83	35	37.6	39	41.9	19	20.4
Nicotine e-cigarettes	2.28	18	19.4	31	33.3	44	47.3
Psychological help/support	1.89	40	43.0	23	24.7	30	32.3

**Table 3:** Distinguishing the correct definition of the harm reduction concept in smoking from incorrect ones.

Harm reduction is:	*N*	%
An approach aiming to reduce the adverse effects of nicotine use	21	22.6
An approach aiming to reduce the negative effects of tobacco smoking, without requiring abstinence	53	57.0
An approach aiming to non-smokers so that they never start smoking	4	4.3
A strategy used to persuade smokers to give up their habit right away	6	6.5
I do not know	9	9.7

**Table 4:** Distinguishing nicotine replacement therapy (NRT) options^a^.

	No		Yes	
	*N*	%	*N*	%
Nicotine patches	3	3.2	90	96.8
Nicotine chewing gum	5	5.4	88	94.6
Heated tobacco products	86	92.5	7	7.5
E-cigarettes	14	15.1	79	84.9
Oral nicotine spray	27	29.0	66	71.0
Nicotine pouches/snus (for nicotine absorption through the buccal mucosa)	47	50.5	46	49.5

^a^Respondents could select multiple options.

**Table 5:** Time spent discussing smoking cessation during a typical consultation/visit.

	*N*	%
Less than 1 min	29	31.2
Between 2 and 5 min	48	51.6
More than 5 min	10	10.8
I do not discuss with patients the need to quit smoking	6	6.5

**Table 6:** Responsibility for providing and implementing smoking cessation according to participants’ opinions^a^.

	No		Yes	
	*N*	%	*N*	%
Trained nurses	23	24.7	70	75.3
Family doctor	14	15.1	79	84.9
Psychiatrist/addictologist	29	31.2	64	68.8
Public health specialist	25	26.9	68	73.1
Other	89	95.7	4	4.3

^a^Respondents could select multiple options.

Questions in a Knowledge theme were a mix of single-answer and multiple-answer formats. For example, one question asked respondents to identify the criteria for defining a smoker, with multiple response options reflecting different smoking histories and intensities (e.g., based on lifetime cigarette consumption, current smoking habits, and frequency of smoking). Suggested correct answers, as indicated in the Supplementary file 1, were provided to participants upon completing the survey. Questions were constructed, and correct answers were drawn from references primarily sourced from systematic reviews published in the Cochrane Database of Systematic Reviews, public health authorities such as the CDC and Health Canada, intergovernmental organizations including the WHO and European Parliament, as well as peer-reviewed publications and institutional guidelines known for methodological rigor and empirical transparency. All the references used for Knowledge theme are listed in a table in Supplementary file 2.

Questions on Attitudes and self-assessed experiences and practices in smoking cessation were measured using a seven-point Likert scale (e.g., *Rate the importance and function of a family doctor in smoking cessation of their patients from 1 = not at all to 7 = completely*). Some questions on self-assessed experiences and practices varied in format, including multiple-choice and binary (yes/no) responses. For instance, participants were asked to estimate the time spent discussing smoking cessation during a typical consultation, with response options ranging from less than 1 min to more than 5 min. Additionally, some questions assessed familiarity with key concepts, such as harm reduction, through yes/no responses (e.g., *Are you familiar with the concept of harm reduction?*).

The demographics of the participants were assessed based on nicotine products used, personal tobacco smoking status, and type of formal training received on smoking cessation.

All questions were formulated by the authors, and the scientific evidence for interpreting correct and incorrect answers is provided in Supplementary file 1, following each question that measures knowledge.

### Ethical considerations

The study protocol was approved by University of Zagreb, School of Medicine, Ethical Board (protocol code: Reg. no.: 380–59–10,106-23-111/108, Class: 641–01/23–02/01, date of approval June 25th 2023). Participants provided electronic informed consent. Anonymization was ensured by omitting age/gender data while maintaining population-level demographic context (79.5% female, average age 31).

### Sample

An online survey was conducted from July 3 to 30, 2023, on a population of 161 respondents. The population are participants of the Postgraduate Specialist Study (PGS) in family medicine at the Faculty of Medicine, University of Zagreb. A convenience sample was used within this population, meaning that participation was voluntary and based on accessibility during the study period. Selection of the population of interest was based on two assumptions. First, family medicine residents, after completing the PGS, should possess the latest insights, knowledge, and attitudes about methods of smoking cessation, which they should be able to advise their patients competently and confidently upon completing specialist training and returning to their home institutions or clinics. Second, due to the reach and specificity of the population cared for by family doctors, the fact that they are the first contact doctors, and their strong influence on changing harmful habits, they represent a group of experts who must identify at-risk individuals and timely and correctly intervene. The survey was conducted over 4 weeks, with the first 2 weeks during the PGS and the remaining two immediately after its completion.

Considering that the population was small, and to maximize the protection of participants’ privacy and minimize the chance of triangulating certain sociodemographic parameters, data on gender and age were not collected. The reason behind the additional efforts to preserve anonymity is that the study also measured knowledge, and based on previous scientific insights, we expected a relatively low level of general knowledge about smoking cessation.

Although data on the gender and age of participants in the sample were not collected, these parameters are known at the population level (of the full list of PGS attendees). The population was predominantly female (79.5%), with an average age of 31 years.

### Analytical methods

A descriptive analysis of frequencies and percentage of responses was carried out, and mean was used as a distribution measure. The data analysis was conducted using the IBM Statistics 26 software package. Incomplete forms were not collected; therefore, no missing data were present in this study.

## Results

The online survey was accessed by 93 PDS participants out of a total of 161, which is a response rate of 57.76%.

Of the ninety-three respondents, 71% (66 respondents) identified as non-smokers. 91.4% of respondents stated that they had never been educated about methods for quitting smoking, and 87.1% of them answered that they were willing to undergo additional training for advising smoking cessation (71% prefer group education or workshops, 55.9% are interested in webinars, and 35.5% for web content/brochures and individual training).

As shown in Table 1, 62.4% of respondents believe that nicotine inhalation causes malignant diseases, while 93.5% believe that inhalation of products resulting from the combustion of various additives in tobacco products, and products of tobacco combustion itself (94.6%), contribute to the development of malignant diseases. 60.2% of respondents believe that the inhalation of combustion products of cigarette paper contributes to the onset of malignant diseases, and an equal percentage believe the same for the inhalation of flavors (menthol, forest fruit, etc.) added to cigarettes.

When asked about the speed of smoking cessation 25.8% of respondents believe that there is no significant difference between abrupt and gradual smoking cessation, while 40.9% believe that abrupt cessation is a better method than gradual smoking cessation. A positive contribution of physical activity to smoking cessation and maintenance of non-smoking was recognized by 89.2% of respondents, while mindfulness methods (dialectical behavioral therapy, yoga, etc.) were considered a significant contribution to smoking cessation by 62.4% of respondents. Non-smoking schools or group counseling were not considered the best way to quit smoking by 24.7% of respondents, while 67.7% believe that the use of mobile applications can help in long-term smoking cessation. From the pharmacotherapeutic options offered in the survey for smoking cessation, 76.3% of respondents would choose Nicorette®, 46.2% varenicline, 35.5% bupropion, while 16.5% of respondents would not prescribe medication therapy at all to assist in smoking cessation.

Respondents were asked to rate three methods of smoking cessation from the most successful to the least successful. Results are shown in Table 2. Nicotine replacement therapy (NRT) was ranked as the most successful method for smoking cessation, followed by psychological support, and the use of nicotine e-cigarettes in third place.

The effectiveness of available/official/recommended methods for smoking cessation was assessed by respondents as weakly effective (44/93, 47.3%), effective (39/93, 41.9%), very effective (8/93, 8.6%), and ineffective (2/93, 2.2%).

75.3% of respondents had heard of the concept of harm reduction, but only 57% recognized the exact definition of harm reduction among the offered answers (Table 3).

In order to test the respondents’ knowledge on Nicotine Replacement Therapy (NRT) they were asked to recognize the options that are considered NRT (Table 4). 7.5% of respondents incorrectly categorized tobacco heating devices as nicotine replacement therapy (NRT), 84.9% categorized e-cigarettes as NRT, and 49.5% categorized nicotine pouches/snus as NRT.

When asked “Can those attempting to quit smoking, to prevent relapse, use nicotine replacement therapy products as long as necessary?,” 33.3% of respondents said that NRT should use for as long as necessary, 44.1% answered negative, and 22.6% do not know if NRT can be used indefinitely.

In everyday clinical practice, 51.6% of respondents stated that they spend between two and five minutes on smoking cessation counseling per consultation or smoker examination, with 31.2% of them spending less than 1 min, and 6.5% not discussing smoking cessation with patients (Table 5).

For 84.9% of respondents, the healthcare professional responsible for implementing and ensuring smoking cessation is the family doctor, while 75.3% believe that this is the role of specially educated nurses, and 73.1% of respondents believe that this is the role of public health specialists or psychiatrists (68.8%) (Table 6).

## Discussion

This study aimed to assess attitudes, knowledge, and behaviors regarding smoking cessation among family medicine residents using the novel SMOKE AKAT questionnaire. Findings revealed critical gaps: 91.4% lacked formal education on cessation methods, 62.4% incorrectly associated nicotine with cancer, and 84.9% misclassified e-cigarettes as nicotine replacement therapy (NRT). These deficiencies persist despite 87.1% of residents expressing interest in further training, underscoring systemic educational shortcomings. The disconnect between residents’ perceived role in smoking cessation (84.9% believing family doctors should lead interventions) and their preparedness to fulfill this role raises concerns about the quality of patient counseling. Family physicians’ strategic position in tobacco cessation is well-documented, with evidence showing brief clinician interventions can increase quit rates by two-thirds. The smoking prevalence of 30% among Croatian family medicine residents, though higher than rates typically reported (the prevalence of smoking among physicians is approximately 21%), is not unusual in an international perspective (31). Large multi-country studies show that physician smoking behavior is strongly shaped by local social norms, institutional culture, occupational stress levels, and the perceived social acceptability of tobacco use. In several world regions—including parts of Asia, Southern and Eastern Europe, and the Middle East—physician smoking prevalence frequently reaches or surpasses 30% (32). Such patterns are often associated with gender distribution, heavy workload, weak institutional tobacco control policies, and insufficient emphasis on tobacco education in medical training (33). Crucially, higher smoking rates among physicians are consistently linked to barriers in providing cessation counseling: those who smoke are less likely to advise patients on quitting, often perceive themselves as less credible, and tend to have diminished influence on patient receptiveness when delivering cessation advice. Cultural professional identity, belief in patient agency or futility, role-modeling, and system-level supports or incentives all interface to determine the likelihood of bringing up, persisting with, and personalizing cessation advice (34). In addition, structural barriers—including time constraints (51.6% allocating ≤5 min per consultation) and knowledge gaps—hinder effective implementation. The misconception that nicotine itself causes cancer may lead to inappropriate harm reduction counseling, particularly given 84.9% misclassifying e-cigarettes as NRT. This confusion mirrors broader challenges in distinguishing evidence-based cessation therapies from emerging products, compounded by rapid market innovations. Recent studies confirm that even brief discussions with physicians can increase cessation rates by approximately two-thirds, highlighting the significant impact of physician intervention (35, 36). Moreover, The World Health Organization’s 2024 clinical treatment guidelines emphasize the importance of healthcare provider-delivered behavioral support alongside pharmacological treatments in comprehensive tobacco cessation strategies (37). Despite this recognized role, family physicians continue to face substantial barriers to providing effective smoking cessation counseling. According to Fiore and colleagues, while initial steps like asking about smoking status and advising cessation are common, subsequent steps such as assessing readiness to quit, providing assistance, and arranging follow-up are frequently neglected (38). A 2024 study among military family physicians found that time constraints (62.6%), lack of supporting staff (34.3%), and inadequate resources (48%) were major barriers to promoting smoking cessation and integrating it with preventive services like lung cancer screening (39). These findings align with our results, indicating a persistent gap between the expected role of family physicians and their preparedness to fulfill this role effectively.

The absence of a standardized smoker definition complicates intervention targeting. While CDC’s 100-cigarette threshold provides operational clarity, its clinical applicability remains debated, particularly for light or intermittent smokers (40). This definitional ambiguity may contribute to inconsistent counseling practices, as seen in 6.5% of residents not discussing cessation at all. Croatia’s medical education system exacerbates these challenges, with 91.4% of residents reporting no formal training—a deficit requiring urgent curriculum reforms addressing nicotine pharmacology and harm reduction principles. According to GOLD guidelines for the treatment of chronic obstructive pulmonary disease (COPD), exposure to tobacco smoke is cited as the main risk factor for disease development, and smoking cessation is recommended (41).

In the Republic of Croatia, education on smoking and smoking cessation methods for healthcare professionals is not implemented in the curricula of medical and related faculties but depends on individual engagement. According to the results of our research, about 90% of respondents have never been educated, while at the same time, the same percentage wants education on smoking cessation methods. The importance of quality education of healthcare workers on this topic is more important than ever because new products, with or without tobacco, and with or without nicotine, are constantly emerging on the market. The identified knowledge gaps about fundamental aspects of smoking cessation among family medicine residents raise significant concerns about the quality of cessation counseling provided to patients. The misconception that nicotine itself causes cancer (held by 62.4% of our respondents) may lead to inappropriate counseling regarding NRT and harm reduction strategies. This finding resonates with international evidence suggesting that healthcare providers’ knowledge about nicotine and tobacco differs substantially from scientific consensus (42). The widespread misclassification of e-cigarettes as NRT (84.9% of respondents) further indicates confusion about the distinction between harm reduction tools and evidence-based cessation therapies. This knowledge deficit could result in suboptimal treatment recommendations and missed opportunities for effective intervention.

The limited time allocated to smoking cessation counseling (2–5 min per visit) reported by over half of the residents reflects the practical constraints of primary care settings but may be insufficient for addressing complex addiction issues. Research indicates that more intensive interventions with multiple contacts are generally more effective than brief interventions, suggesting that current practice patterns may be inadequate (12). The finding that 87.1% of residents express interest in additional training presents an opportunity to address these knowledge gaps and improve the quality of cessation counseling. This study introduces a valuable new assessment tool—the “SMOKE AKAT” questionnaire—which can be utilized to evaluate smoking cessation knowledge and attitudes among healthcare professionals beyond our sample. The identified knowledge gaps provide clear direction for medical education curriculum developers seeking to improve smoking cessation training. Recent educational innovations, such as the longitudinal smoking cessation counseling course based on the 5A model implemented at the University of Würzburg, demonstrate promising approaches to addressing these educational needs (36). Their findings that practical implementation with real patients significantly improved students’ counseling confidence and competence support our recommendation for enhanced experiential learning opportunities in smoking cessation counseling.

The scientific significance of this research extends beyond identifying educational gaps. By documenting specific misconceptions about nicotine, NRT, and harm reduction, our findings contribute to the broader understanding of barriers to evidence-based smoking cessation care. These insights can inform tailored educational interventions that specifically address common misconceptions and knowledge deficits among family medicine residents. Based on our findings, several recommendations can be made to improve smoking cessation training and practice in family medicine. Improvements should prioritize multi-level interventions. Medical curricula must integrate evidence-based cessation training using frameworks like the 5A model, emphasizing practical skill development through clinical rotations and simulated patient interactions (36, 43). Healthcare systems should allocate dedicated consultation time for cessation counseling and implement electronic health record prompts to standardize documentation. This includes allocating appropriate time for cessation counseling, developing team-based approaches that involve nurses and other staff, and ensuring adequate reimbursement for cessation services. The integration of smoking cessation counseling with other preventive services, such as lung cancer screening, represents an efficient approach that should be further developed and promoted (39). Family medicine practices should implement systematic protocols for identifying smokers, documenting smoking status, and providing consistent cessation support. Electronic health record prompts, clinical decision support tools, and quality improvement initiatives focused on smoking cessation metrics can help standardize and enhance cessation care (44). Continuing education programs must address specific knowledge gaps, p particularly regarding nicotine’s health effects, proper classification of cessation aids, and evidence-based harm reduction strategies, while policymakers should leverage tools like SMOKE AKAT for competency assessments and targeted interventions.

Research should continue to evaluate the effectiveness of different educational approaches and practice models for enhancing smoking cessation counseling in primary care. Longitudinal studies that track both physician knowledge/attitudes and patient cessation outcomes are particularly needed to inform best practices.

### Limitations and future directions

This pilot study has several limitations that should be addressed in future research. The current study provides valuable insights into smoking cessation knowledge gaps among family medicine residents but has limited external validity due to its convenience sampling strategy, geographic specificity, lack of demographic diversity, and cross-sectional design. Addressing these limitations through broader sampling methods, demographic analysis, longitudinal designs, and cross-context validation would strengthen the generalizability of future research findings. The cross-sectional design cannot capture changes in knowledge and attitudes over time. Future studies should include longitudinal follow-up to assess how knowledge and practices evolve throughout residency and into independent practice. Additionally, comparing the findings with those from practicing family physicians could provide insights into whether experience compensates for formal education gaps. While we implemented anonymous data collection to reduce social desirability bias, residual measurement bias may persist due to self-reporting.

The framework for developing the smoking cessation knowledge, attitudes and behaviors questionnaire presented limitations in content validity due to the absence of cognitive debriefing interviews with the target population. By relying on literature reviews and author expertise, the questionnaire development lacked to incorporate family medicine physicians’ perspectives during item refinement, leaving possible ambiguities in some constructs unaddressed. This omission underscores the necessity of integrating iterative debriefing phases in future instrument development to align expert-defined constructs with the linguistic and conceptual frameworks of end-users.

The questionnaire’s development focused on educational barriers—specifically healthcare professionals’ limited training in smoking cessation interventions, nicotine replacement therapies, and harm reduction principles—as primary constraints to implementing effective strategies. However, the instrument does not account for additional determinants spanning individual, organizational, and systemic levels—including practitioners’ motivational drivers, workplace resource availability, regulatory constraints, and social-environmental influences—that collectively shape clinical capacity to deliver evidence-based cessation care. Some of the mentioned determinants should be integrated in the future instrument development.

Future research should also explore the relationship between family physicians’ knowledge about smoking cessation and actual patient outcomes. Studies linking physician knowledge scores with patient quit rates would provide valuable evidence regarding the clinical significance of the identified knowledge gaps. Furthermore, investigating patients’ perspectives on the smoking cessation counseling they receive from family physicians would offer complementary insights to enhance our understanding of effective cessation support.

As this is the first pilot testing of the questionnaire and no scale-based constructs were used, it was not possible to assess construct validity. A dedicated study aimed at validating the SMOKE AKAT questionnaire, specifically assessing its reliability and construct validity, represents a valuable direction for future research.

## Conclusion

The study identifies systemic gaps in smoking cessation education and practice among family medicine residents, with implications for public health policy and medical training. High rates of nicotine-related misconceptions and product misclassification highlight the need for curriculum reforms addressing emerging tobacco products and evidence-based cessation methods. Residents’ willingness to engage in training presents an opportunity to implement standardized educational modules combining theoretical knowledge with practical counseling skills. Structural reforms should align clinical practice with WHO guidelines, ensuring adequate time and resources for cessation counseling.

The SMOKE AKAT questionnaire emerges as a valuable tool for identifying knowledge deficiencies and evaluating educational interventions, with potential applications across healthcare systems pursuing smoke-free targets. The SMOKE AKAT serves dual purposes as both a research instrument and a practical training supplement to both national protocols and the WHO clinical treatment guidelines for adult tobacco cessation. Its capacity to benchmark provider competencies enables three critical functions: establishing baseline performance metrics, guiding the development of tailored educational programs, and monitoring competency enhancements following training interventions.

## Data Availability

The data that support the findings of this study are available from the corresponding author upon reasonable request.

## References

[1] Marteau TM Hollands GJ Fletcher PC. Changing human behavior to prevent disease: the importance of targeting automatic processes. Science. (1979) 337:1492–5. doi: 10.1126/science.1226918, PMID: 22997327

[2] Langford R Bonell CP Jones HE Pouliou T Murphy SM Waters E. The WHO health promoting school framework for improving the health and well-being of students and their academic achievement. Cochrane Database Syst Rev. (2014) 2014:CD008958. doi: 10.1002/14651858.CD008958.pub2, PMID: 24737131 PMC11214127

[3] Van Ryzin MJ Fosco GM Dishion TJ. Family and peer predictors of substance use from early adolescence to early adulthood: an 11-year prospective analysis. Addict Behav. (2012) 37:1314–24. doi: 10.1016/j.addbeh.2012.06.020, PMID: 22958864 PMC3459354

[4] Catalano RF Fagan AA Gavin LE Greenberg MT Irwin CE Jr Ross DA. Worldwide application of prevention science in adolescent health. Lancet. (2012) 379:1653–64. doi: 10.1016/s0140-6736(12)60238-4, PMID: 22538180 PMC4398056

[5] Foxcroft DR Tsertsvadze A. Universal family-based prevention programs for alcohol misuse in young people. Cochrane Database Syst Rev. (2011):CD009308. doi: 10.1002/14651858.cd009308, PMID: 21901733

[6] Krist AH Davidson KW Mangione CM Barry MJ Cabana M Caughey AB. Behavioral counseling interventions to promote a healthy diet and physical activity for cardiovascular disease prevention in adults with cardiovascular risk factors: US preventive services task force recommendation statement. JAMA. (2020) 324:2069–75. doi: 10.1001/jama.2020.21749, PMID: 33231670

[7] Starfield B Shi L Macinko J. Contribution of primary care to health systems and health. Milbank Q. (2005) 83:457–502. doi: 10.1111/j.1468-0009.2005.00409.x, PMID: 16202000 PMC2690145

[8] Anczak JD Nogler RA. Tobacco cessation in primary care: maximizing intervention strategies. Clin Med Res. (2003) 1:201–16. doi: 10.3121/cmr.1.3.201, PMID: 15931310 PMC1069046

[9] Vogt F Hall S Marteau TM. General practitioners’ and family physicians’ negative beliefs and attitudes towards discussing smoking cessation with patients: a systematic review. Addiction. (2005) 100:1423–31. doi: 10.1111/j.1360-0443.2005.01221.x, PMID: 16185204

[10] European Network for Smoking and Tobacco Prevention. Guidelines 2020 English. Accessed Feb 28, 2025. (2020). Available online at: https://ensp.network/wp-content/uploads/2020/10/guidelines_2020_english_forprint.pdf.

[11] Tucker JS Stucky BD Edelen MO Shadel WG Klein DJ. Healthcare provider counseling to quit smoking and patient desire to quit: the role of negative smoking outcome expectancies. Addict Behav. (2018) 85:8–13. doi: 10.1016/j.addbeh.2018.05.008, PMID: 29793182 PMC9284996

[12] Lindson N Pritchard G Hong B Fanshawe TR Pipe A Papadakis S. Strategies to improve smoking cessation rates in primary care. Cochrane Database Syst Rev. (2021) 9:CD011556. doi: 10.1002/14651858.CD011556.pub2, PMID: 34693994 PMC8543670

[13] Lua Y How C Ng C. Smoking cessation in primary care. Singapore Med J. (2024) 65:38–44. doi: 10.4103/singaporemedj.SMJ-2022-034, PMID: 38212983 PMC10863739

[14] Geller AC Powers CA. Medical education teaching smoking cessation in U.S. medical schools: A long way to go. 9; (2007). Available online at: www.virtualmentor.org 10.1001/virtualmentor.2007.9.1.medu1-0701 23217665

[15] Krist AH Davidson KW Mangione CM Barry MJ Cabana M Caughey AB. Interventions for tobacco smoking cessation in adults, including pregnant persons: US preventive services task force recommendation statement. JAMA. (2021) 325:265–79. doi: 10.1001/jama.2020.25019, PMID: 33464343

[16] Vogt F Hall S Hankins M Marteau TM. Evaluating three theory-based interventions to increase physicians’ recommendations of smoking cessation services. Health Psychol. (2009) 28:174–82. doi: 10.1037/a0013783, PMID: 19290709

[17] Hall S Vogt F Marteau TM. A short report: survey of practice nurses’ attitudes towards giving smoking cessation advice. Fam Pract. (2005) 22:614–6. doi: 10.1093/fampra/cmi082, PMID: 16055470

[18] World Health Organization Western Pacific Region. Providing tobacco cessation support and protocols. Geneva, Switzerland: WHO: (2020).

[19] United States Public Health Service Office of the Surgeon General; National Center for Chronic Disease Prevention and Health Promotion (US) Office on Smoking and Health.. Smoking Cessation: A Report of the Surgeon General. Washington (DC): US Department of Health and Human Services; (2020).

[20] Caponnetto P Polosa R. Common predictors of smoking cessation in clinical practice. Respir Med. (2008) 102:1182–92. doi: 10.1016/j.rmed.2008.02.017, PMID: 18586479

[21] Rigotti NA. Training future physicians to deliver tobacco cessation treatment. J Gen Intern Med. (2016) 31:144–6. doi: 10.1007/s11606-015-3560-7, PMID: 26747628 PMC4720659

[22] WHO Report on the Global Tobacco Epidemic, (2019). Geneva: World Health Organization.

[23] Boopathirajan R Muthunarayanan L. Awareness, attitude and use of tobacco among medical students in Chennai. J Lifestyle Med. (2017) 7:27–34. doi: 10.15280/jlm.2017.7.1.27, PMID: 28261558 PMC5332118

[24] Daudt AW Alberg AJ Prola JC Fialho L Petracco A Wilhelms A. A first step incorporating smoking education into a Brazilian medical school curriculum: results of a survey to assess the cigarette smoking knowledge, attitudes, behaviour, and clinical practices of medical students. J Addict Dis. (1999) 18:19–29. doi: 10.1300/J069v18n01_03, PMID: 10234560

[25] Carson KV Verbiest MEA Crone MR Verbiest ME Brinn MP Esterman AJ. Training health professionals in smoking cessation. Cochrane Database Syst Rev. (2012) 2012:CD000214. doi: 10.1002/14651858.CD000214.pub2, PMID: 22592671 PMC10088066

[26] Gautier S Cloppet A Mir S Duville C Morvillers JM Simzac AB. Knowledge, attitudes and practices of primary healthcare professionals regarding smoking and smoking cessation among the elderly in France. Tob Prev Cessat. (2023) 9:1. doi: 10.18332/tpc/173401, PMID: 37915359 PMC10616976

[27] Oreskovic S Percac-Lima S Ashburner JM Tiljak H Rifel J Klemenc Ketiš Z. Cytisine Versus Varenicline for Smoking Cessation in a Primary Care Setting: A Randomized Non-inferiority Trial. Nicotine Tob Res. (2023) 25:1547–55. doi: 10.1101/2021.07.03.21259600 37291049

[28] Čivljak M Ačkar L Puljak L. The knowledge, attitudes and behaviors of hospital nurses on smoking cessation interventions: a cross-sectional study. BMC Nurs. (2023) 22:228. doi: 10.1186/s12912-023-01394-7, PMID: 37394472 PMC10316570

[29] Schneider NK Vražić H Ljubičić Đ. Smoking and basic medical education in Croatia. Eur Respir J. (2006) 28:1. doi: 10.1007/s00038-008-7005-516816343

[30] Čivljak M Čivljak R Kuzman I Grgić A Šember D Belak Kovačević S. Feasibility of the multimodal smoking cessation intervention during hospitalization with six-month follow-up post-discharge: a pilot study. Acta Clin Croat. (2022) 61:273–83. doi: 10.20471/acc.2022.61.02.14, PMID: 36818939 PMC9934034

[31] Besson A Tarpin A Flaudias V Brousse G Laporte C Benson A. Smoking prevalence among physicians: a systematic review and Meta-analysis. Int J Environ Res Public Health. (2021) 18:13328. doi: 10.3390/ijerph182413328, PMID: 34948936 PMC8705497

[32] Zafar M. Prevalence of smoking and associated risk factors among medical professionals in hospitals of Karachi, Pakistan. Int J Prev Med. (2014) 5:457–62. PMID: 24829733 PMC4018594

[33] Zong Q Li H Jiang N Gong Y Zheng J Yin X. Prevalence and determinants of smoking behavior among physicians in emergency department: a national cross-sectional study in China. Front Public Health. (2022) 10:980208. doi: 10.3389/fpubh.2022.980208, PMID: 36324466 PMC9620959

[34] Gollust SE Schroeder SA Warner KE. Helping smokers quit: understanding the barriers to utilization of smoking cessation services. Milbank Q. (2008) 86:601–27. doi: 10.1111/j.1468-0009.2008.00536.x, PMID: 19120982 PMC2690372

[35] Stead LF Buitrago D Preciado N Sanchez G Hartmann-Boyce J Lancaster T. Physician advice for smoking cessation. Cochrane Database Syst Rev. (2013) 2013:CD000165. doi: 10.1002/14651858.CD000165.pub4, PMID: 23728631 PMC7064045

[36] Ruck J Tiedemann E Sudmann J Kübler A Simmenroth A. Evaluating the longitudinal effectiveness of a smoking cessation counselling course based on the 5A model for medical students in family medicine placement. GMS J Med Educ. (2025) 42:Doc10. doi: 10.3205/zma001734, PMID: 40395954 PMC12086244

[37] World Health Organization. WHO clinical treatment guideline for tobacco cessation in adults. Geneva: (2024). 38954657

[38] Fiore MC Bailey WC Cohen SJ (1996) Smoking Cessation: Clinical Practice Guideline No. 18 US Department of Health and Human Services, Public Health Service, Agency for Health Care Policy and Research

[39] Owens DK Davidson KW Krist AH Barry MJ Cabana M Caughey AB. Primary Care Interventions for Prevention and Cessation of Tobacco Use in Children and Adolescents: US Preventive Services Task Force Recommendation Statement. JAMA. (2020) 323:1590–1598. doi: 10.1001/jama.2020.4679 32343336

[40] Centers for Disease Control and Prevention. National Health Interview Survey (NHIS) - adult tobacco use - glossary. CDC. Accessed March 23, 2023. (2017). Available online at: https://www.cdc.gov/nchs/nhis/tobacco/tobacco_glossary.htm.

[41] Agustí A Celli BR Criner GJ Halpin D Anzueto A Barnes P. Global initiative for chronic obstructive lung disease 2023 report: GOLD executive summary. Eur Respir J. (2023) 61:2300239. doi: 10.1183/13993003.00239-2023, PMID: 36858443 PMC10066569

[42] Patwardhan S Rose JE. Overcoming barriers to disseminate effective smoking cessation treatments globally. Drugs Alcohol Today. (2020) 20:235–47. doi: 10.1108/DAT-01-2020-0001

[43] Hartmann-Boyce J Livingstone-Banks J Ordóñez-Mena JM Fanshawe TR Lindson N Freeman SC. Behavioural interventions for smoking cessation: an overview and network meta-analysis. Cochrane Database Syst Rev. (2021) 1:CD013229. doi: 10.1002/14651858.CD013229.pub2, PMID: 33411338 PMC11354481

[44] Boyle R Solberg L Fiore M. Use of electronic health records to support smoking cessation. Cochrane Database Syst Rev. (2014) 2014:CD008743. doi: 10.1002/14651858.CD008743.pub3, PMID: 25547090 PMC7173728

[45] Centers for Disease Control and Prevention. Tobacco-related surveillance: tobacco terminology glossary. Available online at: https://www.cdc.gov/nchs/nhis/tobacco/tobacco_glossary.htm [Accessed March 23, 2023]

[46] Health Canada. Tobacco use statistics: terminology. (2025). Available online at: https://www.canada.ca/en/health-canada/services/health-concerns/tobacco/research/tobacco-use-statistics/terminology.html [Accessed March 30, 2023]

[47] Hecht SS. Tobacco smoke carcinogens and lung cancer. JNCI J Natl Cancer Inst. (1999) 91:1194–210. doi: 10.1093/jnci/91.14.1194, PMID: 10413421

[48] Ussher MH Faulkner GEJ Angus K Hartmann-Boyce J Taylor AH. Exercise interventions for smoking cessation. Cochrane Database Syst Rev. (2019) 2019:CD002295. doi: 10.1002/14651858.CD002295.pub6, PMID: 31684691 PMC6819982

[49] Nicorette Australia. Quickmist nicotine spray. Available online at: https://www.nicorette.com.au/products/quickmist-nicotine-spray [Accessed March 30, 2023]

[50] Nicorette Australia. Products. (2024). Available online at: https://www.nicorette.com.au/products [Accessed March 30, 2023]

[51] American Cancer Society. Guide to quitting smoking: nicotine replacement therapy. (2024). Available online at: https://www.cancer.org/healthy/stay-away-from-tobacco/guide-quitting-smoking/nicotine-replacement-therapy.html [Accessed March 30, 2023]

[52] European Parliament and Council. Directive 2014/40/EU on the approximation of the laws, regulations and administrative provisions of the member states concerning the manufacture, presentation and sale of tobacco and related products. (2014). Available online at: https://health.ec.europa.eu/system/files/2016-11/dir_201440_en_0.pdf [Accessed May 25, 2023]

[53] Medicines and Healthcare products Regulatory Agency. Nicotine replacement therapy and harm reduction. Drug Safety Update. (2010) 3:7–6.

[54] Stead LF Carroll AJ Lancaster T. Group behaviour therapy programmes for smoking cessation. Cochrane Database Syst Rev. (2017) 3:CD001007. doi: 10.1002/14651858.CD001007.pub3, PMID: 28361497 PMC6464070

[55] Whittaker R McRobbie H Bullen C Rodgers A Gu Y Dobson R. Mobile phone text messaging and app-based interventions for smoking cessation. Cochrane Database Syst Rev. (2019) 10:CD006611. doi: 10.1002/14651858.CD006611.pub5, PMID: 31638271 PMC6804292

[56] Centers for Disease Control and Prevention. Tips from former smokers®: QuitSTART app. (2024). Available online at: https://www.cdc.gov/tobacco/campaign/tips/quit-smoking/quitstart-app/index.html [Accessed March 30, 2023]

[57] Smokefree.gov. Smartphone apps. Available online at: https://smokefree.gov/tools-tips/apps [Accessed March 30, 2023]

[58] Healthline. The best quit smoking apps of 2023. (2020). Available online at: https://www.healthline.com/health/quit-smoking/top-iphone-android-apps#my-quit-buddy (Accessed March 30, 2023)

[59] Jackson S Brown J Norris E Livingstone-Banks J Hayes E Lindson N. Mindfulness for smoking cessation. Cochrane Database Syst Rev. (2022) 2022:CD013696. doi: 10.1002/14651858.CD013696.pub2, PMID: 35420700 PMC9009295

[60] Hartmann-Boyce J Lindson N Butler AR McRobbie H Bullen C Begh R. Electronic cigarettes for smoking cessation. Cochrane Database Syst Rev. (2022) 11:CD010216. doi: 10.1002/14651858.CD010216.pub7, PMID: 36384212 PMC9668543

[61] Hughes JR Keely J Naud S. Shape of the relapse curve and long-term abstinence among untreated smokers. Arch Intern Med. (2004) 99:29–38. doi: 10.1111/j.1360-0443.2004.00540.x, PMID: 14678060

[62] Gardois P Booth A Goyder E Ryan T. Health promotion interventions for increasing stroke awareness in ethnic minorities: a systematic review of the literature. BMC Public Health. (2014) 14:409. doi: 10.1186/1471-2458-14-409, PMID: 24775404 PMC4019964

[63] Stead LF Perera R Bullen C Mant D Hartmann-Boyce J Cahill K. Nicotine replacement therapy for smoking cessation. Cochrane Database Syst Rev. (2012) 11:CD000146. doi: 10.1002/14651858.CD000146.pub4, PMID: 23152200

[64] Hatsukami DK Carroll DM. Tobacco harm reduction: past history, current controversies and a proposed approach for the future. Prev Med. (2020) 140:106099. doi: 10.1016/j.ypmed.2020.106099, PMID: 32335031 PMC7581601

[65] World Health Organization. WHO model list of essential medicines, 22nd list (2021). Available online at: https://www.who.int/publications/i/item/WHO-MHP-HPS-EML-2021.02 [Accessed March 30, 2023]

[66] International Agency for Research on Cancer. IARC monographs on the evaluation of carcinogenic risks to humans, volume 100E: personal habits and indoor combustions (2012). Available online at: https://monographs.iarc.who.int/wp-content/uploads/2018/06/mono100E-6.pdf [Accessed March 30, 2023] PMC4781577 23193840

