# Supplementary material for: Navigating smoking cessation in healthcare: a pilot study of the SMOKE AKAT questionnaire among family medicine residents
Source: Front Public Health. 2025 Oct 9;13:1471124. doi: 10.3389/fpubh.2025.1471124 (PMC12546155; doi:10.3389/fpubh.2025.1471124)
Supplement: Supplementary file 1 [file Data_Sheet_1.pdf]

Attitudes and **K**nowledge Accessing **T**ool on **Smok**ing Cessation Methods (SMOKE AKAT)

Iva Petričušić, Ljiljana Ćenan, Hana Brborović, Ognjen Brborović

Smoking cessation reduces the risk of morbidity and mortality from cardiovascular, cerebrovascular, pulmonary, and malignant diseases. The purpose of this intervention study is to learn about the attitudes and assess the knowledge of family medicine residents in the Republic of Croatia.

By participating in this anonymous survey, you consent to data processing in accordance with the provisions of the Personal Data Protection Act.

1. **A smoker is a person who (multiple-choice question)?**

A has smoked 100 cigarettes in their lifetime and currently does not smoke

B is 18 years old and has smoked 100 cigarettes in their lifetime so far

C smokes up to one pack of cigarettes per week

D has been smoking only 2 cigarettes with their morning coffee for years

E has smoked only 5 packs of cigarettes in the last 10 years and only occasionally lights up

Suggestion correct answers B, C, D, E

<u>Learning opportunity for participants who would like to know more:</u>

45. Centers for Disease Control and Prevention. Tobacco-related surveillance: tobacco terminology glossary. Available at: https://www.cdc.gov/nchs/nhis/tobacco/tobacco_glossary.htm [Accessed March 23, 2023]

46. Health Canada. Tobacco use statistics: terminology. Available at: https://www.canada.ca/en/health-canada/services/health-concerns/tobacco/research/tobacco-use-statistics/terminology.html [Accessed March 30, 2023]

2. **A non-smoker is a person who (multiple-choice question)?**

A has never smoked

B has smoked only 50 cigarettes in their lifetime

C occasionally lights up while having coffee

D smoked for 10 years and has been using only e-cigarettes for a year

E does not buy cigarettes but sometimes asks for a cigarette in social situations

Suggestion correct answers A, B,

Learning opportunity for participants who would like to know more:

45. Centers for Disease Control and Prevention. Tobacco-related surveillance: tobacco terminology glossary. Available at: https://www.cdc.gov/nchs/nhis/tobacco/tobacco_glossary.htm [Accessed March 23, 2023]

46. Health Canada. Tobacco use statistics: terminology. Available at: https://www.canada.ca/en/health-canada/services/health-concerns/tobacco/research/tobacco-use-statistics/terminology.html [Accessed March 30, 2023]

3. **What is the percentage of smokers in the population under your or your mentor's care (if you do not have your own GP office yet)?**

A _______(enter the percentage)

B I have never checked/asked for this information

4. **Rate the importance and function of a family doctor in smoking cessation of their patients (from 1=not at all to 7=completely).**

1234567

5. **How much time do you spend talking to a smoker about quitting smoking during a usual consultation/visit?**

A less than 1 minute

B between 2 and 5 minutes

C more than 5 minutes

D I do not discuss with patients the need to quit smoking

6. **Select all answers that you believe influence the occurrence of smoking-related cancers. (Multiple-choice question):**

A inhalation of substances produced by the combustion of tobacco

B inhalation of substances produced by the combustion of cigarette paper

C inhalation of flavors (menthol/forest fruits etc.) added to cigarettes

D inhalation of various additives to tobacco used in cigarette preparation

E inhalation of nicotine

Suggestion correct answers A, B, C, D

Learning opportunity for participants who would like to know more:

47. Hecht SS. Tobacco smoke carcinogens and lung cancer. JNCI: Journal of the National Cancer Institute (1999) 91:1194–1210. doi: 10.1093/jnci/91.14.1194

7. **Abrupt smoking cessation is a better method than gradual withdrawal from smoking.**

A Yes

B No

C there is no difference

Suggestion correct answers C

Learning opportunity for participants who would like to know more:

43. Hartmann-Boyce J, Livingstone-Banks J, Ordóñez-Mena JM, Fanshawe TR, Lindson N, Freeman SC, et al. Behavioural interventions for smoking cessation: an overview and network meta-analysis. Cochrane Database Syst Rev (2021) 1:CD013229. doi: 10.1002/14651858.CD013229.pub2

8. **Choose the medications you would prescribe to a patient as pharmacotherapy options for smoking cessation. (Multiple-choice question)**

A Wellbutrin ®

B Varenicline ®

C Nicorette ®

D Zomig ®

E I would not prescribe medications as an option for smoking cessation

Suggestion correct answers A, B, C

Learning opportunity for participants who would like to know more:

42. Patwardhan S, Rose JE. Overcoming barriers to disseminate effective smoking cessation treatments globally. Drugs and Alcohol Today (2020) 20:235-247. doi: 10.1108/DAT-01-2020-0001

9. **Can physical activity contribute to achieving and maintaining smoking cessation?**

A Yes

B No

C slightly contributes

Suggestion correct answers C

Learning opportunity for participants who would like to know more:

48. Ussher MH, Faulkner GEJ, Angus K, Hartmann-Boyce J, Taylor AH. Exercise interventions for smoking cessation. Cochrane Database Syst Rev (2019) 10:CD002295. doi: 10.1002/14651858.CD002295.pub6

10. Choose the options that are classified as nicotine replacement therapy (NRT) (multiple-choice question)

A nicotine patches

B nicotine chewing gum

C heated tobacco products

D e-cigarettes

E oral nicotine spray

F nicotine pouches/snus (for nicotine absorption through the buccal mucosa)

Suggestion correct answers A, B, E

Learning opportunity for participants who would like to know more:

49. Nicorette Australia. Quickmist nicotine spray. Available at: https://www.nicorette.com.au/products/quickmist-nicotine-spray [Accessed March 30, 2023]

50. Nicorette Australia. Products. Available at: https://www.nicorette.com.au/products [Accessed March 30, 2023]

51. American Cancer Society. Guide to quitting smoking: nicotine replacement therapy. Available at: https://www.cancer.org/healthy/stay-away-from-tobacco/guide-quitting-smoking/nicotine-replacement-therapy.html [Accessed March 30, 2023]

42. Patwardhan S, Rose JE. Overcoming barriers to disseminate effective smoking cessation treatments globally. Drugs and Alcohol Today (2020) 20:235-247. doi: 10.1108/DAT-01-2020-0001

52. European Parliament and Council. Directive 2014/40/EU on the approximation of the laws, regulations and administrative provisions of the Member States concerning the manufacture, presentation and sale of tobacco and related products. Available at: https://health.ec.europa.eu/system/files/2016-11/dir_201440_en_0.pdf [Accessed May 25, 2023]

11. Can those attempting to quit smoking, to prevent relapse, use nicotine replacement therapy products as long as necessary?

A Yes

B No

C Don’t know

Suggestion correct answers A

Learning opportunity for participants who would like to know more:

53. Medicines and Healthcare products Regulatory Agency. Nicotine replacement therapy and harm reduction. Drug Safety Update (2010) 3:6. Available at: https://www.gov.uk/drug-safety-update/nicotine-replacement-therapy-and-harm-reduction [Accessed March 30, 2023]

12. Group counseling or “schools of smoking cessation” are the best method for smoking cessation.

A Yes

B No

Suggestion correct answers B

Learning opportunity for participants who would like to know more:

54. Stead LF, Carroll AJ, Lancaster T. Group behaviour therapy programmes for smoking cessation. Cochrane Database Syst Rev (2017) 3:CD001007. doi: 10.1002/14651858.CD001007.pub3

13. Using mobile applications can increase the proportion of people who quit smoking long-term.

A Yes

B No

Suggestion correct answers A

Learning opportunity for participants who would like to know more:

55. Whittaker R, McRobbie H, Bullen C, Rodgers A, Gu Y, Dobson R. Mobile phone text messaging and app-based interventions for smoking cessation. Cochrane Database Syst Rev (2019) 10:CD006611. doi: 10.1002/14651858.CD006611.pub5

56. Centers for Disease Control and Prevention. Tips From Former Smokers®: QuitSTART App. Available at: https://www.cdc.gov/tobacco/campaign/tips/quit-smoking/quitstart-app/index.html [Accessed March 30, 2023]

57. Smokefree.gov. Smartphone apps. Available at: https://smokefree.gov/tools-tips/apps [Accessed March 30, 2023]

58. Healthline. The best quit smoking apps of 2023. Available at: https://www.healthline.com/health/quit-smoking/top-iphone-android-apps#my-quit-buddy [Accessed March 30, 2023]

14. To what extent do mindfulness methods (yoga, Dialectical Behavior Therapy DBT, Acceptance and Commitment Therapy AC etc.) contribute to smoking cessation?

A do not contribute

B significantly contribute

C extremely contribute

D No clear benefits or evidence

Suggestion correct answers D

Learning opportunity for participants who would like to know more:

59. Jackson S, Brown J, Norris E, Livingstone-Banks J, Hayes E, Lindson N. Mindfulness for smoking cessation. Cochrane Database Syst Rev (2022) 4:CD013696. doi: 10.1002/14651858.CD013696.pub2

15. **According to your own opinion, rank the methods of smoking cessation from the most successful to the least successful.**

A nicotine replacement therapy NRT____

B nicotine e-cigarettes____

C psychological help/support___

Suggestion correct answers A2 B1 C3

Learning opportunity for participants who would like to know more:

60. Hartmann-Boyce J, Lindson N, Butler AR, McRobbie H, Bullen C, Begh R, et al. Electronic cigarettes for smoking cessation. Cochrane Database Syst Rev (2022) 11:CD010216. doi: 10.1002/14651858.CD010216.pub7

16. **How effective do you consider the existing official/available/recommended tools for smoking cessation (nicotine patches and gum, non-smoking schools etc.)?**

A very effective (>10%)

B effective (5-10%)

C low effectiveness (2-

5%) D ineffective (<2%)

Suggestion correct answers B

Learning opportunity for participants who would like to know more:

61. Hughes JR, Keely J, Naud S. Shape of the relapse curve and long-term abstinence among untreated smokers. Arch Intern Med (2004) 164:659-660. doi: 10.1001/archinte.164.6.659

62. Bauld L, Bell K, McCullough L, Richardson L, Greaves L. Health promotion interventions for increasing stroke awareness in ethnic minorities: a systematic review of the literature. BMC Public Health (2013) 13:409. doi: 10.1186/1471-2458-13-409

63. Stead LF, Perera R, Bullen C, Mant D, Hartmann-Boyce J, Cahill K, et al. Nicotine replacement therapy for smoking cessation. Cochrane Database Syst Rev (2012) 2:CD000146. doi: 10.1002/14651858.CD000146.pub4

54. Stead LF, Carroll AJ, Lancaster T. Group behaviour therapy programmes for smoking cessation. Cochrane Database Syst Rev (2017) 3:CD001007. doi: 10.1002/14651858.CD001007.pub3

60. Hartmann-Boyce J, Lindson N, Butler AR, McRobbie H, Bullen C, Begh R, et al. Electronic cigarettes for smoking cessation. Cochrane Database Syst Rev (2022) 11:CD010216. doi: 10.1002/14651858.CD010216.pub7

17. Rate your OWN success in applying smoking cessation methods (from 1=not at all to 7=completely). If you would never recommend a method, choose *I do not conduct/recommend*

A	individual and group counseling	1234567	I do not conduct/recommend
B	pharmacotherapy	1234567	I do not conduct/recommend
C	hypnosis	1234567	I do not conduct/recommend
D	nicotine replacement therapy	1234567	I do not conduct/recommend
E	e-cigarettes	1234567	I do not conduct/recommend

<u>Learning opportunity for participants who would like to know more:</u>

43. Hartmann-Boyce J, Livingstone-Banks J, Ordóñez-Mena JM, Fanshawe TR, Lindson N, Freeman SC, et al. Behavioural interventions for smoking cessation: an overview and network meta-analysis. Cochrane Database Syst Rev (2021) 1:CD013229. doi: 10.1002/14651858.CD013229.pub2

54. Stead LF, Carroll AJ, Lancaster T. Group behaviour therapy programmes for smoking cessation. Cochrane Database Syst Rev (2017) 3:CD001007. doi: 10.1002/14651858.CD001007.pub3

60. Hartmann-Boyce J, Lindson N, Butler AR, McRobbie H, Bullen C, Begh R, et al. Electronic cigarettes for smoking cessation. Cochrane Database Syst Rev (2022) 11:CD010216. doi: 10.1002/14651858.CD010216.pub7

18. What sources of information do you use to providing smoking cessation to the patients? (Multiple-choice question)

A Public media or television

B Internet

C Other healthcare workers

DMedical conferences

EScientific publications

FI do not need additional sources to inform myself on that behalf

19. Did you receive any formal training in providing smoking cessation?

A Yes

BNo

20. In your own opinion, which healthcare worker should be most responsible for providing and implementing smoking cessation? *(multiple-choice question)

A Trained nurses

B Family doctor

C psychiatrist / addictologist

DPublic health specialist

EOther ___________

21. Are you willing to receive formal training for providing smoking cessation?

A Yes

BNo

22. If YES, what kind of formal training would you like to receive for smoking cessation? (multiple-choice question)

A Flyers, papers, web contents

B Group education - workshops

CIndividual training

DWebinars

E Other ______

23. Are you familiar with the concept of harm reduction?

A Yes

BNo

24. Which of the following sentences best describes the *harm reduction concept* in smoking?

A Harm reduction is an approach aiming to reduce the adverse effects of nicotine use.

B Harm reduction is an approach aiming to reduce the negative effects of tobacco smoking, without requiring abstinence.

C Harm reduction is an approach aiming to non-smokers so that they never start smoking.

DHarm reduction is a strategy used to persuade smokers to give up their habit right away

EI do not know

Suggestion correct answers B

<u>Learning opportunity for participants who would like to know more:</u>

64. Hatsukami DK, Carroll DM. Tobacco harm reduction: Past history, current controversies and a proposed approach for the future. Prev Med (2020) 140:106099. doi: 10.1016/j.ypmed.2020.106099

25. Rate the importance and function of a family doctor in smoking cessation of their patients (from 1=not at all to 7=completely).

1234567

26. "*Nicotine in tobacco products does not cause cancer. It is addictive. The harm from smoking is due to the thousands of toxic chemicals in the smoke, including over a hundred carcinogens. Therefore, I feel confident in prescribing nicotine replacement therapy (NRT) to smokers in adequate doses and for long enough, so as to ensure complete smoking cessation and prevent future relapse*." To what extent do you agree with the above statement?

A Do not agree. Nicotine is carcinogenic, and there are far better smoking cessation methods than nicotine products.

B Somewhat agree. Nicotine is not carcinogenic; however, it should not be used for too long, even as an NRT, as that would mean that patients are still dependent on nicotine, and dependence is bad.

C Totally agree. Nicotine is the key addictive component of tobacco and cigarette smoke. However, it is not carcinogenic. Therefore, prescribing NRT for smoking cessation for as long as needed to prevent relapse should be fine.

Suggestion correct answers C

Learning opportunity for participants who would like to know more:

65. World Health Organization. WHO model list of essential medicines, 22nd list (2021). Available at: https://www.who.int/publications/i/item/WHO-MHP-HPS-EML-2021.02 [Accessed March 30, 2023]

66. International Agency for Research on Cancer. IARC monographs on the evaluation of carcinogenic risks to humans, Volume 100E: personal habits and indoor combustions (2012). Available at: https://monographs.iarc.who.int/wp-content/uploads/2018/06/mono100E-6.pdf [Accessed March 30, 2023]

27. What is your personal tobacco smoking status?

A non smoker

B former smoker

Csmoker

28. Do you use other nicotine products?

A nicotine patches

B nicotine chewing gums

Cnicotine pouches / snus

Delectronic cigarettes

Eheated tobacco products

29. If you are interested in receiving written materials used in the creation of the questionnaire, other materials on smoking cessation methods, or notifications about the organization of workshops/courses on smoking cessation methods, please enter your email address at the following link.

0/1 (if interested, the designation is 1)
