# Supplementary material for: Navigating smoking cessation in healthcare: a pilot study of the SMOKE AKAT questionnaire among family medicine residents
Source: Front Public Health. 2025 Oct 9;13:1471124. doi: 10.3389/fpubh.2025.1471124 (PMC12546155; doi:10.3389/fpubh.2025.1471124)
Supplement: Supplementary file 2 [file Data_Sheet_2.pdf]

Supplemetary file 2. Question-Source Validity Alignment

KNOWLEDGE THEME	
QUESTION NUMBER	SOURCE
1	Centers for Disease Control and Prevention. Tobacco-related surveillance: tobacco terminology glossary. Available at: https://www.cdc.gov/nchs/nhis/tobacco/tobacco_glossary.htm [Accessed March 23, 2023] (41), Health Canada. Tobacco use statistics: terminology. Available at: https://www.canada.ca/en/health-canada/services/health-concerns/tobacco/research/tobacco-use-statistics/terminology.html [Accessed March 30, 2023] (42)
2	Centers for Disease Control and Prevention. Tobacco-related surveillance: tobacco terminology glossary. Available at: https://www.cdc.gov/nchs/nhis/tobacco/tobacco_glossary.htm [Accessed March 23, 2023] (41), Health Canada. Tobacco use statistics: terminology. Available at: https://www.canada.ca/en/health-canada/services/health-concerns/tobacco/research/tobacco-use-statistics/terminology.html [Accessed March 30, 2023] (42)
6	Hecht SS. Tobacco smoke carcinogens and lung cancer. JNCI: Journal of the National Cancer Institute (1999) 91:1194–1210. doi: 10.1093/jnci/91.14.1194 (43)
7	Hartmann-Boyce J, Livingstone-Banks J, Ordóñez-Mena JM, Fanshawe TR, Lindson N, Freeman SC, et al. Behavioural interventions for smoking cessation: an overview and network meta-analysis. Cochrane Database Syst Rev (2021) 1:CD013229. doi: 10.1002/14651858.CD013229.pub2 (44)
8	Patwardhan S, Rose JE. Overcoming barriers to disseminate effective smoking cessation treatments globally. Drugs and Alcohol Today (2020) 20:235-247. doi: 10.1108/DAT-01-2020-0001 (45)
9	Ussher MH, Faulkner GEJ, Angus K, Hartmann-Boyce J, Taylor AH. Exercise interventions for smoking cessation. Cochrane Database Syst Rev (2019) 10:CD002295. doi: 10.1002/14651858.CD002295.pub6 (46)
10	Nicorette Australia. Quickmist nicotine spray. Available at: https://www.nicorette.com.au/products/quickmist-nicotine-spray [Accessed March 30, 2023] (47), Nicorette Australia. Products. Available at: https://www.nicorette.com.au/products [Accessed March 30, 2023] (48), American Cancer Society. Guide to quitting smoking: nicotine replacement therapy. Available at: https://www.cancer.org/healthy/stay-away-from-tobacco/guide-quitting-smoking/nicotine-replacement-therapy.html [Accessed March 30, 2023] (49), Patwardhan S, Rose JE. Overcoming barriers to disseminate effective smoking cessation treatments globally. Drugs and Alcohol Today (2020) 20:235-247. doi: 10.1108/DAT-01-2020-0001 (45), European Parliament and Council. Directive 2014/40/EU on the approximation of the laws, regulations and administrative provisions of the Member States concerning the manufacture, presentation and sale of tobacco and related products. Available at: https://health.ec.europa.eu/system/files/2016-11/dir_201440_en_0.pdf [Accessed May 25, 2023] (50)
11	Medicines and Healthcare products Regulatory Agency. Nicotine replacement therapy and harm reduction. Drug Safety Update (2010) 3:6. Available at: https://www.gov.uk/drug-safety-update/nicotine-replacement-therapy-and-harm-reduction [Accessed March 30, 2023] (51)
12	Stead LF, Carroll AJ, Lancaster T. Group behaviour therapy programmes for smoking cessation. Cochrane Database Syst Rev (2017) 3:CD001007. doi: 10.1002/14651858.CD001007.pub3 (52)

13	Whittaker R, McRobbie H, Bullen C, Rodgers A, Gu Y, Dobson R. Mobile phone text messaging and app-based interventions for smoking cessation. Cochrane Database Syst Rev (2019) 10:CD006611. doi: 10.1002/14651858.CD006611.pub5 (53), Centers for Disease Control and Prevention. Tips From Former Smokers®: QuitSTART App. Available at: https://www.cdc.gov/tobacco/campaign/tips/quit-smoking/quitstart-app/index.html [Accessed March 30, 2023] (54), Smokefree.gov. Smartphone apps. Available at: https://smokefree.gov/tools-tips/apps [Accessed March 30, 2023] (55), Healthline. The best quit smoking apps of 2023. Available at: https://www.healthline.com/health/quit-smoking/top-iphone-android-apps#my-quit-buddy [Accessed March 30, 2023] (56)
14	Jackson S, Brown J, Norris E, Livingstone-Banks J, Hayes E, Lindson N. Mindfulness for smoking cessation. Cochrane Database Syst Rev (2022) 4:CD013696. doi: 10.1002/14651858.CD013696.pub2 (57)
15	Hartmann-Boyce J, Lindson N, Butler AR, McRobbie H, Bullen C, Begh R, et al. Electronic cigarettes for smoking cessation. Cochrane Database Syst Rev (2022) 11:CD010216. doi: 10.1002/14651858.CD010216.pub7 (58)
16	Hughes JR, Keely J, Naud S. Shape of the relapse curve and long-term abstinence among untreated smokers. Arch Intern Med (2004) 164:659-660. doi: 10.1001/archinte.164.6.659 (59), Bauld L, Bell K, McCullough L, Richardson L, Greaves L. Health promotion interventions for increasing stroke awareness in ethnic minorities: a systematic review of the literature. BMC Public Health (2013) 13:409. doi: 10.1186/1471-2458-13-409 (60), Stead LF, Perera R, Bullen C, Mant D, Hartmann-Boyce J, Cahill K, et al. Nicotine replacement therapy for smoking cessation. Cochrane Database Syst Rev (2012) 2:CD000146. doi: 10.1002/14651858.CD000146.pub4 (61), Stead LF, Carroll AJ, Lancaster T. Group behaviour therapy programmes for smoking cessation. Cochrane Database Syst Rev (2017) 3:CD001007. doi: 10.1002/14651858.CD001007.pub3 (52), Hartmann-Boyce J, Lindson N, Butler AR, McRobbie H, Bullen C, Begh R, et al. Electronic cigarettes for smoking cessation. Cochrane Database Syst Rev (2022) 11:CD010216. doi: 10.1002/14651858.CD010216.pub7 (58)
24	Hatsukami DK, Carroll DM. Tobacco harm reduction: Past history, current controversies and a proposed approach for the future. Prev Med (2020) 140:106099. doi: 10.1016/j.ypmed.2020.106099 (62)
26	World Health Organization. WHO model list of essential medicines, 22nd list (2021). Available at: https://www.who.int/publications/i/item/WHO-MHP-HPS-EML-2021.02 [Accessed March 30, 2023] (63), International Agency for Research on Cancer. IARC monographs on the evaluation of carcinogenic risks to humans, Volume 100E: personal habits and indoor combustions (2012). Available at: https://monographs.iarc.who.int/wp-content/uploads/2018/06/mono100E-6.pdf [Accessed March 30, 2023] (64)
